# Vitamin D: Are We Ready to Supplement for Breast Cancer Prevention and Treatment?

**DOI:** 10.1155/2013/483687

**Published:** 2013-02-26

**Authors:** Katherine D. Crew

**Affiliations:** ^1^Department of Medicine, Division of Hematology/Oncology, College of Physicians and Surgeons, Columbia University, New York, NY 10032, USA; ^2^Department of Epidemiology, Mailman School of Public Health, Columbia University, New York, NY 10032, USA; ^3^Herbert Irving Comprehensive Cancer Center, Columbia University, New York, NY 10032, USA

## Abstract

Vitamin D deficiency is a potentially modifiable risk factor that may be targeted for breast cancer prevention and treatment. Preclinical studies support various antitumor effects of vitamin D in breast cancer. Numerous observational studies have reported an inverse association between vitamin D status, including circulating 25-hydroxyvitamin D (25(OH)D) levels, and breast cancer risk. The relationship between vitamin D and mammographic density, a strong predictor of breast cancer risk, remains unclear. Studies analyzing the link between genetic polymorphisms in vitamin D pathway genes and breast cancer incidence and prognosis have yielded inconsistent results. Vitamin D deficiency among breast cancer patients has been associated with poorer clinical outcomes and increased mortality. Despite a number of clinical trials of vitamin D supplementation, the efficacy, optimal dosage of vitamin D, and target blood level of 25(OH)D for breast cancer prevention have yet to be determined. Even with substantial literature on vitamin D and breast cancer, future studies need to focus on gaining a better understanding of the biologic effects of vitamin D in breast tissue. Despite compelling data from experimental and observational studies, there is still insufficient data from clinical trials to make recommendations for vitamin D supplementation for breast cancer prevention or treatment.

## 1. Introduction

Breast cancer confers significant morbidity and mortality among women in the United States. Due to the magnitude of this disease, considerable research effort has been directed toward identifying breast cancer risk factors to target for prevention. However, relatively few modifiable lifestyle and environmental factors have been associated with reduced breast cancer risk. Chemoprevention refers to altering the carcinogenesis process with a drug intervention. The selective estrogen receptor modulators (SERMs), tamoxifen [1] and raloxifene [2], and aromatase inhibitor (AI), exemestane [3], have been shown to reduce breast cancer incidence. These antiestrogens are the only drugs that have been approved by the USA Food and Drug Administration for breast cancer prevention in high-risk population; however, uptake has been poor in the prevention setting. Due to serious toxicities associated with SERMs, namely, endometrial cancer and thromboembolic disease, and chronic toxicities of AIs, such as hot flashes, arthralgias, and osteoporosis, they have not gained widespread acceptance in the primary prevention setting. In addition, these antiestrogens do not lower the incidence of more aggressive estrogen receptor- (ER-) negative breast cancers, which account for about one-third of all breast tumors and are associated with a poorer prognosis compared with ER-positive breast cancer. Therefore, a priority in breast cancer chemoprevention includes developing safe and tolerable agents for potential chronic use that are effective against ER-negative tumors. There is an urgent need to develop agents which modulate nonendocrine biochemical pathways in breast carcinogenesis.

The earliest observations linking vitamin D with cancer came from ecological studies [4]. Since ultraviolet B (UVB) light is essential for cutaneous production of vitamin D, sunlight exposure may be a surrogate for vitamin D levels. One study suggested a potential link between increasing rates of certain cancers and geographic latitude, allowing researchers to postulate that decreased sunlight exposure, leading to less endogenous production of vitamin D, may in fact increase rates of malignancy [5]. Other ecological studies have associated increased sunlight exposure with low breast cancer incidence and mortality [6–10]. These studies laid the foundation for examining the hypothesis that vitamin D deficiency increases cancer risk and mortality, including breast cancer. 

 As a result of heightened interest in this association, there have been a number of recent observational studies, as well as clinical trials of vitamin D supplementation, examining the relationship between vitamin D status and breast cancer. Vitamin D deficiency is often defined as a serum 25-hydroxyvitamin D (25(OH)D) less than 20 ng/mL or 50 nmol/L (1 ng/mL = 2.5 nmol/L). This has become a shared concern among physicians, many of whom now routinely screen for vitamin D deficiency and/or recommend supplementation both in healthy women and breast cancer patients. This interest prompted a 2010 Institute of Medicine (IOM) report addressing vitamin D supplementation [11]. The recommended dietary allowance (RDA) of vitamin D was increased from 400 IU to 600 IU daily for persons 70 years and younger and 800 IU daily for persons over 70 years, intakes corresponding to serum 25(OH)D greater than 20 ng/mL (or 50 nmol/L). In addition, the upper safety limit in healthy individuals was raised from 2000 IU to 4000 IU daily. However, the IOM also raised concerns about negative health effects for circulating 25(OH)D levels above 50 ng/mL (or 125 nmol/L) [11]. After a thorough review of the literature, they concluded that there was insufficient evidence to recommend vitamin D supplementation for cancer prevention or treatment (http://www.IOM.edu/vitaminD). In this paper, we evaluate the current knowledge about the anticancer properties of vitamin D and its association with breast cancer, with an emphasis on the literature published within the past 5 years. We conclude by discussing future directions in this field.

## 2. Vitamin D Metabolism

**Figure 1:**
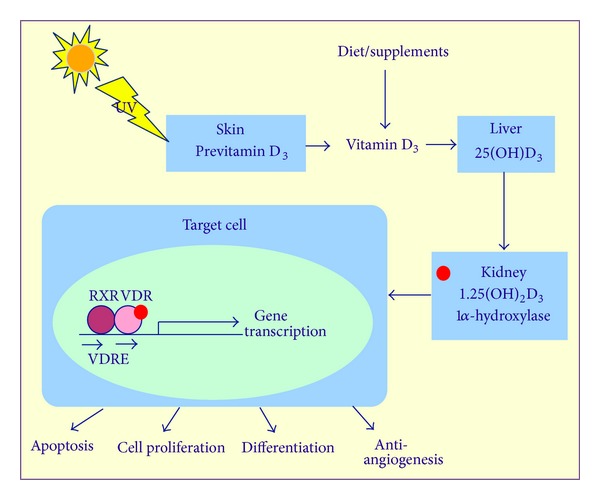
Vitamin D metabolism.

Vitamin D does not meet the strict definition of a vitamin, an essential nutrient that the body cannot synthesize in sufficient quantities. It represents a fat-soluble prohormone that must be modified within the body to produce its active metabolites (Figure 1) [12]. It can exist in two forms: vitamin D_3_ (cholecalciferol), which is metabolized in the skin by the action of ultraviolet B (UVB) light on a cholesterol product which is synthesized in the skin (7-dehydrocholesterol), or vitamin D_2_ (ergocalciferol), which is obtained via plant-based dietary sources. Foods of animal origin rich in vitamin D_3_ include egg yolk, dairy fat, liver, and oily fish [4]. Modest amounts of vitamin D come from food sources, but the majority of vitamin D (up to 90%) comes from endogenous production in the skin. As a result, wide variability in vitamin D status occurs due to differences in geographic location, season, sun avoidance behaviors, sunscreen use, increasing age and skin pigmentation (due to decreased skin synthesis of vitamin D), obesity (due to fat sequestration of vitamin D), and other lifestyle factors [13]. Vitamin D deficiency is surprisingly common, especially among the elderly, blacks, and residents of northern climates [14].

Whether derived from the diet or synthesized in the skin, the precursor form of cholecalciferol/ergocalciferol is then transferred bound to vitamin D binding protein (DBP) within the bloodstream to the liver, where it is hydroxylated by mitochondrial and microsomal 24-hydroxylase (encoded by *CYP24A1)* to 25-hydroxyvitamin D (25(OH)D) or calcidiol. Although 25(OH)D has low biological activity, it is the major circulating form and serves as an integrated measure of vitamin D from all sources such as diet, supplements, and sunlight exposure [15]. This product is taken to the kidneys, where it is hydroxylated by mitochondrial 1*α*-hydroxylase (encoded by *CYP27B1*) into 1,25-dihydroxyvitamin D (1,25(OH)_2_D) or calcitriol, the hormonally active form [16]. Many extrarenal tissues also express 1*α*-hydroxylase and, therefore, have the enzymatic machinery to locally activate vitamin D, which acts in a paracrine and autocrine manner in these tissues [17]. Circulating 25(OH)D is the substrate for conversion to 1,25(OH)_2_D in target tissues by 1*α*-hydroxylase and may be the limiting factor in local activation of vitamin D [18]. In addition, cancer cells express 1*α*-hydroxylase; therefore, raising serum 25(OH)D levels may be a useful chemopreventive and treatment strategy to allow intratumor synthesis of calcitriol [19].

Calcitriol is further hydroxylated by 24-hydroxylase (encoded by *CYP24A1*), creating two less active forms, 24,25(OH)_2_D and 1*α*,24,25(OH)_2_D, which are then excreted primarily in the feces [20]. In target tissues, including cancer cells, calcitriol induces expression of 24-hydroxylase [21]. Therefore, coadministration of calcitriol with an agent that inhibits 24-hydroxylase (e.g., the soy isoflavone, genistein) may be a rational combination strategy for cancer prevention and treatment [22]. Using vitamin D analogs which resist 24-hydroxylation is an alternative strategy for cancer therapy [23].

The vitamin D receptor (VDR) is a ligand-dependent transcription factor that is part of a nuclear receptor super-family. The receptor is comprised of two zinc finger structures with a characteristic DNA-binding domain and a carboxy-terminal ligand-binding domain [24]. When bound to its ligand, calcitriol (1,25(OH)_2_D), VDR dimerizes with the retinoid X receptor (RXR), causing a conformational change that allows the heterodimer to translocate into the nucleus, where it binds to vitamin D response elements (VDRE) in promoter regions, allowing for transcriptional regulation of target genes, such as *p21, p27, c-fos, *and *c-myc* [16, 25]. VDR regulates a wide range of cellular mechanisms central to cancer development, such as apoptosis, cell proliferation, differentiation, angiogenesis, and metastasis [26]. In genomic profiling experiments conducted in breast cancer cell lines cultured with calcitriol (activated vitamin D) and feeding studies of cholecalciferol (parent vitamin D) in mouse models of breast cancer, vitamin D was shown to impact signaling pathways in differentiation, alter metabolism, remodel of extracellular matrix, and innate immunity [27]. In fact, almost 3% of the human genome is thought to be either directly or indirectly regulated by vitamin D [16].

## 3. Preclinical Studies of Vitamin D

Apart from its classical actions on bone and mineral metabolism, vitamin D also has diverse biological effects relevant to carcinogenesis. Calcitriol and various vitamin D analogs have been tested in *in vitro *studies of cancer cell lines. Interestingly, these studies demonstrated that cancer cells undergo certain physiologic changes, which decrease their susceptibility to calcitriol. Malignant cells have decreased intracellular levels of 1*α*-hydroxylase (the activating enzyme encoded by *CYP27B1*) compared to normal cells, which decreases intracellular calcitriol production. Furthermore, there is increased breakdown of calcitriol in tumor cells, causing resistance to the antitumor effects of vitamin D [30]. The vitamin D receptor (VDR) itself can also become altered and restricted to the nucleus, decreasing its binding to the predominately cytoplasmic calcitriol [31]. Colon, breast, and lung cancer have all demonstrated downregulation of expression of VDR when compared to normal cells and well-differentiated tumors have shown comparably more VDR expression as measured by immunohistochemistry when compared to their poorly differentiated counterparts [30]. Higher tumor VDR expression has also been correlated with better prognosis in cancer patients [32].

Calcitriol may play an important role in normal mammary development. Animal models to analyze the effect of VDR on cell growth and development include transgenic VDR knockout mice [26]. VDR-deficient mice are born with profound disruption in calcium homeostasis, changes in duodenal calcium absorption and bone mineralization. Even when supplemental calcium is administered, further phenotypic symptoms present themselves, such as growth retardation, uterine hypoplasia, impaired ovarian folliculogenesis, and reproductive dysfunction [31]. Breast tissue from VDR knockout mice has accelerated growth and branching morphogenesis during pubertal development, as well as increased growth in response to exogenous estrogen and progesterone compared to wild-type mice [26]. VDR knockout mice also demonstrate greater rates of carcinogen-induced preneoplastic mammary lesions compared to wild-type mice [26]. Additionally, 1*α*-hydroxylase expression has been localized to mouse mammary epithelial cells, and treatment with a vitamin D analog increases this expression [33]. In studies of nude mice injected with cultured MCF-7 human breast cancer cells, oral and IV calcitriol inhibited growth of breast tumors [34]. Lee et al. also found that vitamin D analogs significantly suppressed tumor growth in mouse xenograft models [35]. In rodent models of carcinogen-induced breast cancer, calcitriol and its analogs demonstrated significant tumor growth inhibition [36]. 

VDR has been implicated in cell cycle arrest, apoptosis, and promotion of differentiation. VDR inhibits cell proliferation via *p21* and *p27*, which act on G0/G1 cell cycle arrest. The receptor modulates *p21 *via direct binding to vitamin D response elements (VDRE) in its promoter region, whereas it induces *p27* via activation of various transcription factors and protein stabilization mechanisms [30]. G1 cell cycle arrest is also affected by VDR via direct induction of GADD45-alpha, a regulator of NF-*κ*B [31]. VDR has also been linked to regulation of apoptosis, as it is transcriptionally upregulated by p53 and has overlapping transcriptional targets. Via the p53 pathway, VDR is able to detect DNA damage and facilitate DNA repair, preventing mutations and promoting appropriate development [31]. VDR can also function independently of p53 by upregulating BAX and BAK (proapoptotic proteins) and downregulating BCL2 and BCL-XL (antiapoptotic proteins) [20]. Calcitriol has been shown to induce the differentiation of both normal and malignant cells [31]. Specific pathways affected include both epidermal growth factor receptor (EGFR) and insulin-like growth factor-1 (IGF-1), causing inhibition of the MAPK and ERK pathways [37]. By promoting differentiation, VDR facilitates normal development and prevents progression to malignancy. 

There is known crosstalk between calcitriol and estrogen receptor (ER) signaling in breast cancer cells. In ER-positive breast cancer cell lines, calcitriol reduced ER expression by direct transcriptional repression of the *ER*α**gene [38, 39]. Calcitriol also downregulates aromatase gene expression, which encodes the enzyme responsible for estrogen conversion from androgen precursors [40]. Calcitriol inhibits aromatase expression by direct transcriptional repression of human breast cancer cells and cultured preadipocytes [40]. Similarly, in *in vivo *experiments, calcitriol decreased aromatase expression in human breast cancer cells and the surrounding mammary adipose tissue [40]. Of note, the effects of vitamin D compounds on breast cancer cells also occur via ER-independent pathways [41, 42].

Another potential mechanism of antitumor activity of vitamin D is the anti-inflammatory effects [43]. Calcitriol regulates the expression of several prostaglandin pathway genes in ER-positive and ER-negative breast cancer cells [40], including decreasing cyclooxygenase-2 (COX-2) and increasing 15-hydroxyprostaglandin dehydrogenase (15-PGDH) levels [44]. Therefore, calcitriol reduced the effects of prostaglandins on proliferation. Given that COX-2 and aromatase expression in breast tumors is tightly coupled [45, 46], calcitriol-mediated reduction in prostaglandins provides an indirect mechanism for downregulation of aromatase expression.

The effects of calcitriol on angiogenesis may be mediated by prostaglandins, which are important proangiogenic factors [43]. Calcitriol and vitamin D analogs also directly inhibited the proliferation of endothelial cells via vascular endothelial growth factor (VEGF), the most potent stimulator of angiogenesis [47, 48]. Calcitriol also inhibited VEGF-induced endothelial cell tube formation *in vitro* [47, 48] and decreased tumor vascularization in mouse xenografts of VEGF-overexpressing cells [49].

To evaluate vitamin D's therapeutic potential, researchers have studied the effects of using calcitriol in combination with other chemotherapeutic agents. Vitamin D can potentiate the effects of certain therapies such as platinum analogs, taxanes, and DNA-intercalating agents. Ma et al. evaluated the combination of vitamin D and cisplatin in a squamous cell carcinoma (SCC) model and found that pretreatment with calcitriol enhanced the effects of cisplatin on induction of apoptosis [50]. Similarly, Chaudhary et al. determined that pretreatment of breast cancer cells *in vitro* with a vitamin D analog enhanced the effects of Adriamycin as well as irradiation by causing apoptosis, reducing clonogenic survival, and decreasing viable cell numbers [51]. Paclitaxel and calcitriol combinations have been studied in SCC and prostate cancer cell lines [52]. Interestingly, many of these antineoplastic agents have been found to inadvertently increase vitamin D levels by decreasing the stability of *CYP24A1* mRNA (encoding the deactivating enzyme, 24-hydroxylase), demonstrating a method by which these agents work synergistically with vitamin D [30].

Together these preclinical studies point to specific mechanisms of action of vitamin D in cancer prevention and treatment.

## 4. Vitamin D Status and Breast Cancer Risk

Some epidemiological data support an inverse association between vitamin D status (including sunlight exposure, dietary and supplement intake, and direct measurement of circulating vitamin D levels) and breast cancer risk, which has resulted in increased interest in the use of vitamin D for breast cancer prevention. In addition to the ecological studies, a significant inverse association was seen between personal ultraviolet B (UVB) exposure and breast cancer risk [53]. 

Among observational studies examining the association between vitamin D intake from diet and supplements, 10 were case-control studies [54–63] and 10 cohort studies [64–73]. A recent meta-analysis found a significant inverse association between high versus low vitamin D intake and breast cancer risk (relative risk (RR) = 0.91, 95% confidence interval (CI) = 0.85–0.97) [74]. One case-control study found no association between vitamin D and calcium intake from food only and breast cancer risk, but supplemental vitamin D intake of >400 IU daily compared to no intake was associated with reduced risk (odds ratio (OR) = 0.76, 95% CI = 0.59–0.98). However, the National Health and Nutrition Examination Survey (NHANES) follow-up study [64] found no association between dietary and supplemental intake of vitamin D and breast cancer, consistent with more recent reports [70–72]. In this study population, frequent recreational and occupational sunlight exposure was inversely related to breast cancer risk. A limitation of these studies is that dietary intake of vitamin D is not a complete measure of vitamin D status and is subject to measurement error. In the French E3N cohort, only in regions with high UVB exposure was high vitamin D intake associated with decreased breast cancer incidence (hazard ratio (HR) = 0.68, 95% CI = 0.54–0.85) [73].

The majority of studies have assessed the effects of dietary and supplemental intake of vitamin D; however, endogenous production is an important source of vitamin D. Circulating 25-hydroxyvitamin D (25(OH)D) concentration correlates with exogenous vitamin D sources (dietary and supplement intake) and endogenous production through sunlight exposure and is considered the best indicator of vitamin D status [13, 75]. Circulating levels of 25(OH)D (calcidiol) and 1,25(OH)_2_D (calcitriol) may be measured to determine vitamin D status. The metabolically active form of vitamin D, 1,25(OH)_2_D, has low serum levels, a short halflife of 4–6 hours, and is tightly regulated by the kidneys to maintain calcium homeostasis. Furthermore, serum 1,25(OH)D_2_D may not reflect the autocrine and paracrine effects of vitamin D in target tissues [76]. 25(OH)D is the metabolite most often quantified due to its long halflife of 3 weeks, which allows it to exist within the serum in concentrations which are three orders of magnitude higher than the serum concentrations of 1,25(OH)_2_D [12]. 

25(OH)D was first measured using a binding protein assay, in which vitamin D binding protein (DBP) was used to measure blood levels of 25(OH)D. The problem with this method was that the DBP was relatively nonspecific as it bound to other vitamin D metabolites, causing overestimation of the levels of vitamin D by 10%–20%. In 1985, a radioimmunoassay (RIA) was developed to measure 25(OH)D; however, it too measured other metabolites and had a similar level of inaccuracy. In the last few years, a more specific RIA has been developed to measure 25(OH)D with 100% specificity. Other methods used to measure 25(OH)D include high performance liquid chromatography (HPLC) and mass spectroscopy, which can distinguish between precursor forms of vitamin D_2_ (ergocalciferol, from plant-based sources) and vitamin D_3_ (cholecalciferol, from animal sources and endogenous production) [76]. 

In a recent meta-analysis of observational studies, an increase in serum 25(OH)D by 20 ng/mL (or 50 nmol/L) was inversely associated with breast cancer risk, with a summary RR = 0.59 (95% CI = 0.48–0.73) for case-control studies, RR = 0.92 (95% CI = 0.82–1.04) for cohort studies, and RR = 0.73 (95% CI = 0.60–0.88) for both study designs combined [77]. Another meta-analysis reported that each 10 ng/mL increase in serum 25(OH)D was associated with decreased breast cancer risk (summary RR = 0.89, 95% CI = 0.81–0.98) [78]. No significant association was seen for circulating 1,25(OH)_2_D levels and breast cancer risk (OR = 0.99, 95% CI = 0.68–1.44) [74].

Recent population-based case-control studies have examined levels of serum and/or plasma 25(OH)D in relation to *in situ* and invasive breast cancer and found a significant dose-dependent inverse association (Table 1). For each 4-5 ng/mL (or 10–12.5 nmol/L) increase in 25(OH)D level, there was about a 7%–12% reduction in the risk of breast cancer [79, 83, 89]. Variations in results between the studies may be due to differences in geographic locations of the study populations, leading to differential blood levels of 25(OH)D. In the German studies, breast cancer cases had a mean serum 25(OH)D concentration of approximately 18 ng/mL (or 45 nmol/L) [79, 83], whereas in the US study (located at a lower latitude), cases had a higher mean blood level of 25(OH)D of 27.1 ng/mL (or 67.8 nmol/L) [89]. In case-control studies, measurement of serum 25(OH)D occurs in individuals already diagnosed with cancer. Therefore, these results need to be interpreted with caution, because behavioral changes after a cancer diagnosis may also influence vitamin D status (e.g., dietary changes, decreased sunlight exposure due to disability or lifestyle changes).

Data from prospective cohort studies have yielded inconsistent results. Two nested case-control studies both found strong inverse associations between vitamin D status and breast cancer risk (RR = 0.52, 95% CI = 0.32–0.85 and RR 0.73, 95% CI = 0.55–0.96, resp.) [86, 88]. The first study in a Danish population found that the inverse association was more pronounced in premenopausal compared to postmenopausal women (RR = 0.38, 95% CI = 0.15–0.97 versus RR = 0.71, 95% CI = 0.38–1.30, resp.) [86]. Three US studies found no association between prediagnostic 25(OH)D blood levels and breast cancer risk [82, 85, 90]. In the Cancer Prevention study II (CPS-II) Nutrition Cohort, blood draws in postmenopausal women occurred on average 3.9 years prior to breast cancer diagnosis [85]. The second study was a nested case-control study of women participating in the Prostate, Lung, Colorectal, and Ovarian Cancer Screening Trial (PLCO), with a similar average time between baseline blood collection and diagnosis [82]. Higher prediagnostic blood levels of 25(OH)D were not associated with lower risk of postmenopausal breast cancer in either of these studies; however, each relied on a single baseline measurement. In the Women's Health Initiative (WHI) cohort, a significant association was seen between lower serum 25(OH)D and higher breast cancer incidence; however, the finding was attenuated after adjustment for body mass index (BMI) and physical activity [90]. In another cohort study from Sweden, a weak nonsignificant inverse association between serum 25(OH)D concentration and breast cancer risk was initially observed, which disappeared after adjustment for known breast cancer risk factors [87]. 

One possible limitation of these prospective cohort studies is the lag time between serum 25(OH)D measurement in archived blood samples and breast cancer diagnosis. As the follow-up period increases from the time of prediagnostic serum 25(OH)D level to cancer diagnosis, the risk estimates tend toward the null [91]. One study found an inverse correlation with serum 25(OH)D and breast cancer risk for follow-up times up to 10 years, but no correlation beyond 10 years [69]. Circulating 25(OH)D is a useful biomarker for measuring an individual's recent exposure to environmental sources of vitamin D (i.e., over the past 3 months) but may not reflect lifetime patterns of sunlight exposure and vitamin D intake. However, more recent data suggests that serum 25(OH)D levels may remain relatively stable over time [92, 93]. In addition, vitamin D may be more effective at combating cancer near the time of diagnosis [94, 95]. To date, there have been no prospective studies evaluating the effect of change in vitamin D level with serial measurements over time on breast cancer risk.

Prior studies have suggested that the association between vitamin D and breast cancer risk may be stronger for premenopausal women than for postmenopausal women. Two cohort studies showed no reduction in breast cancer risk in association with vitamin D status in postmenopausal women [65, 67]. The Nurses' Health study found an inverse association between vitamin D intake and breast cancer risk among premenopausal women, but no association among postmenopausal women [65]. Similarly, the CPS-II Nutrition Cohort observed no association of breast cancer with total vitamin D intake among postmenopausal women [67]. Results from a large population-based case-control study from Ontario suggested that vitamin D status during adolescence when breast development occurs may be most relevant for breast cancer risk [60].

Some studies have demonstrated that the protective effect of vitamin D on breast cancer risk was independent of tumor hormone receptor status [79, 89]. Recent data from the Nurses' Health study suggested an inverse association for hormone receptor-negative, but not hormone receptor-positive tumors [96]. The Iowa Women's Health study found a stronger protective effect of vitamin D supplement use among women with breast cancers that were negative rather than positive for estrogen receptor (ER) or progesterone receptor (PR) status [69]. These findings are intriguing, given that we currently do not have any effective chemopreventive agents for ER-negative breast cancer.

More consistent observational data support an inverse association between serum vitamin D levels and colorectal cancer risk [11, 97, 98]. However, all of these observational studies may be subject to confounding since factors associated with vitamin D deficiency are also linked to higher breast cancer risk, for example, increasing age, obesity (vitamin D may become sequestered in adipose tissue), low physical activity (correlated with less outdoor activities and sunlight exposure), and other lifestyle factors. Despite promising observational data, individual dietary components (e.g., beta carotene, vitamin E, folic acid, and selenium) have not been successful in preventing cancer in randomized controlled trials [99–103]. Therefore, we must await the results of rigorously conducted randomized controlled trials before making broad recommendations for vitamin D supplementation for breast cancer prevention.

## 5. Vitamin D and Mammographic Density

 Mammographic density refers to the relative proportions of radiolucent fat and radiodense epithelial and stromal tissue within the breast, as seen on mammography, and is one of the strongest predictors of breast cancer development [104, 105]. As such, mammographic density may serve as a useful intermediate biomarker for breast cancer risk assessment in investigations of potential preventive strategies for this disease. Women in the highest quartile of mammographic density demonstrate a 4-to-6-fold increase in breast cancer incidence as compared to women of similar age in the lowest quartile, for up to 10 years following measurement [104–107]. In addition to qualitative measurements, mammographic density can now be assessed on a continuous scale using computer-assisted techniques, including dense area (cm^2^) and percent density (dense area divided by total breast area) [108]. Percent density partly accounts for differences in breast size; however, it may underestimate mammographic density in obese women with large amounts of fat. Body mass index (BMI) has less of an impact on dense area; therefore, this may be a more useful measure of mammographic density in overweight and obese women. Effective breast cancer chemopreventive agents, such as tamoxifen, have been shown to significantly reduce mammographic density within 12–18 months of initiation [109]. However, the effects of nonhormonal agents on mammographic density remain uncertain. The validation of modifiable intermediate biomarkers for short-term breast cancer risk assessment is the key to conducting more efficient breast cancer chemoprevention trials.

A recent systematic review, which included fourteen studies, examined the association between vitamin D and mammographic density [110]. Twelve of the studies were cross-sectional [111–122] and two were prospective studies [123, 124]. Vitamin D status was assessed by dietary and supplement intake in nine studies [116–124] and by circulating 25-hydroxyvitamin D (25(OH)D) levels in five studies [111–115]. Furthermore, only four studies considered dense area in addition to percent density as a measure of mammographic density [111, 114, 115, 119], and five studies included populations which were diverse by race and ethnicity [114, 115, 119–121]. 

In the nine studies which assessed dietary intake of vitamin D, five studies reported a significant inverse association between vitamin D and mammographic density [112, 117–119, 122]. When stratified by menopausal status, much of the association was limited to premenopausal women [117–119]. The association between vitamin D intake and mammographic density among premenopausal women was the strongest in women with high insulin-like growth factor-I (IGF-I) and low IGF binding protein-3 (IGFBP-3) levels [118], suggesting that vitamin D may modulate mammographic density and breast cancer risk via IGF signaling. In a substudy of the Women's Health Initiative (WHI), no association was observed between vitamin D or calcium intake and mammographic density among postmenopausal women, but supplemental vitamin D use was associated with lower density in younger women [121]. Two additional studies found no correlation between vitamin D and mammographic density among postmenopausal women [125, 126]. 

More recent studies that assessed serum 25(OH)D in relation to mammographic density have reported null findings [111–115]. One study found no significant relationship between 25(OH)D blood levels and mammographic density, although women in the highest quartile of serum 25(OH)D had the lowest breast density [111]. In a case-control study nested within the Nurses' Health study, no association was found between circulating levels of 25(OH)D and mammographic density in postmenopausal women. However, women in the highest tertile of mammographic density and lowest tertile of plasma 25(OH)D had a 4-fold greater risk of breast cancer than women with the lowest mammographic density and highest plasma 25(OH)D [113]. Data from a multiethnic cohort of premenopausal women [114] and breast cancer survivors [115] did not support an association between circulating vitamin D levels and mammographic density.

A limitation of all of these studies was that the blood collections for serum 25(OH)D and mammograms were not conducted at the same time point (with time intervals varying from 1 to 8 years). Although most studies adjusted for time between blood draws and mammograms, this may not account for the seasonal variation in vitamin D status. Brisson et al. reported synchronized seasonal variations of mammographic density and 25(OH)D blood levels, demonstrating that the lowest breast density was observed in early December, approximately 4 months after peak serum 25(OH)D [112].

Another potential confounder is obesity, which has been positively associated with postmenopausal breast cancer risk [127] and is also inversely related to vitamin D status and mammographic density. The higher prevalence of vitamin D deficiency among overweight and obese individuals may be due to decreased bioavailability of fat-soluble vitamin D and sequestration in adipose tissue [128]. One study estimated that low vitamin D status may contribute up to 40% of the breast cancer risk attributable to high BMI [129]. However, a recent nested case-control study of the National Surgical Adjuvant Breast and Bowel Project-(NSABP-) P1 trial, which randomized high-risk women to 5 years of tamoxifen versus placebo, found that serum 25(OH)D was not an independent predictor of breast cancer risk after adjustment for BMI [130].

No longitudinal studies to date have documented changes in vitamin D status over time in relation to changes in breast density. The WHI trial, which randomized postmenopausal women to calcium plus vitamin D 400 IU daily or placebo, reported no significant difference in mammographic density after a year of supplementation [131]. However, the ratio of mean density comparing calcium and vitamin D supplementation to placebo was 0.67 (95% CI = 0.41–1.07) with ≥80% study drug compliance and no hormone replacement therapy use. These inconsistent results suggest that potential cancer preventive effects of vitamin D may not be mediated by mammographic density.

## 6. Vitamin D Pathway Genetic Polymorphisms and Breast Cancer Risk

Interindividual variability in response to vitamin D supplementation may be partially due to single nucleotide polymorphisms (SNPs) in vitamin-D-related genes. Polymorphisms are subtle DNA sequence variations, which occur commonly in a given population (>1%) and may have functional significance with modest biological effects. A complementary approach to testing the vitamin-D-cancer hypothesis is to study polymorphisms in the vitamin D receptor (VDR), a key mediator of vitamin D activity which is expressed in normal and malignant breast cells [132]. 

The *VDR* gene is comprised of >100 kb with six promoter regions, six alternatively spliced untranslated exons, and eight protein-coding exons [133]. Over 470 SNPs have been reported in the human *VDR* gene, but the restriction fragment length polymorphisms of *Fokl *(rs10735810) on exon 2, *Apal *(rs7975232) and *Bsml *(rs1544410) on intron 8, and *Taql* (rs731236) on exon 9 have been the most frequently studied [134]. Population studies indicate that genetic variations in the *VDR *gene may affect breast cancer risk, particularly in premenopausal women [135, 136]. 

The *FokI* polymorphism is near the 5′-untranslated region (UTR) within the DNA-binding domain and alters the start codon [137]. The variant T allele (also known as *f* allele) results in a protein that is three amino acids longer and less transcriptionally active in *in vitro *studies [138]. For example, a large nested case-control study found a positive association between the *ff *genotype of *FokI* and breast cancer risk [136], whereas eight other studies found no association with this genotype [80, 139–145]. A recent meta-analysis comparing *FokI ff* with *FF* carriers found a significant increase in breast cancer risk (summary OR = 1.30, 95% CI = 1.04–1.61) [146]. However, Abbas et al. found no association between *FokI *genotype and breast cancer risk, regardless of serum 25(OH)D concentration or other *VDR *polymorphisms [80]. Another study found that *FokI *genotype influenced breast cancer risk when accounting for other *VDR *polymorphisms in haplotype combinations [139]. Other upstream elements in the VDR promoter, in linkage disequilibrium with the *FokI *polymorphism, may also be important in determining expression and strength of transactivation. 

*ApaI, BsmI,* and *TaqI* are located near the 3′-UTR of the *VDR* gene and are in strong linkage disequilibrium [147]. Because these polymorphisms do not alter the amino acid sequence of the VDR protein, their functional significance is not well understood. These 3′ region sequence variants may interact differently with other upstream sequences in the *VDR *gene to regulate transcription, translation, or RNA processing [148, 149]. 

Several studies have reported an association between *BsmI bb *genotype and increased breast cancer risk [139, 140, 145, 150, 151]. For example, Trabert et al. reported a 1.5-fold increased breast cancer risk for postmenopausal Caucasian women with the *bb *genotype; however, this association was not observed among African American women [150]. In addition, women who were homozygous variant for the *BsmI *polymorphism (*bb*) had a 4-fold higher risk of developing metastases compared to women with the *BB *genotype [151]. In a recent meta-analysis, *BsmI Bb* and *BB* carriers had a significantly reduced cancer risk at any site among Caucasian populations [146]. Of note, 70% of commonly used breast cancer cell lines had the high-risk *BsmI bb *genotype [152].

The *TT *genotype of the *TaqI *polymorphism has been associated with reduced circulating levels of vitamin D [153–155]. Of ten epidemiologic studies that have assessed the association between the *TaqI* genotype and breast cancer risk, nine observed no association and one observed a positive association with the *T* allele [142–144, 151, 156–161]. However, *TT* genotype in women with high calcium intake was associated with lower breast cancer risk compared to women with the* tt *or *Tt* genotype and low calcium intake [144]. Therefore, dietary factors may influence the association of *VDR *genotypes with breast cancer risk.

In terms of the *ApaI *polymorphism in the *VDR *gene, the *Aa *and *aa *genotypes were associated with about a 1.5-fold increased breast cancer risk [142]. However, other studies have reported conflicting results with the *AA* genotype correlating with an elevated risk of breast cancer [156, 159]. A nested case-control study within the Cancer Prevention study II (CPS-II) Nutrition Cohort examined the association between breast cancer and these four *VDR* SNPs (*FokI, BsmI, ApaI,* and *TaqI*), as well as SNPs in the 24-hydroxylase gene (*CYP24A1*) and vitamin D-binding protein gene (*DBP*) [144]. Although breast cancer incidence was not associated with any genotype evaluated, *BsmI *and *TaqI *polymorphisms were associated with lower risk among women with high calcium intake. Another study found a significant inverse association between a *DBP *(also known as group-specific component (*GC*) gene) polymorphism and breast cancer risk, independent of 25(OH)D blood levels [81]. In addition, no significant associations were observed between vitamin D pathway polymorphisms (*VDR, CYP27B1,* and *DBP* genes) and mammographic density, an intermediate biomarker of breast cancer risk [162].

Few studies have explored the joint association of genetic variation in *VDR *with biomarkers of vitamin D status, such as serum 25(OH)D. A case-control study nested in the Physicians' Health study found a significant interaction between circulating 25(OH)D levels, *VDR FokI *genotype, and prostate cancer risk [163]. Among women with vitamin D deficiency (plasma 25(OH)D <20 ng/mL or <50 nmol/L), the *FokI ff* genotype correlated with reduced risk of breast cancer (OR = 0.49, 95% CI = 0.29–0.81) [164]. This association is counterintuitive as one would expect that the less-active VDR *f* allele [138, 165, 166] would be associated with increased risk. Further research is needed to replicate the differential associations for breast cancer and to clarify the biological mechanisms responsible. 

The role of *VDR *genotype on breast cancer occurrence remains uncertain, but *VDR *polymorphisms may account for interindividual differences in response to vitamin D. Discrepancies in these results among different study populations may be due to ethnic variation in the frequency of *VDR *gene polymorphisms [167, 168]. Potential gene-environment interactions may exist between polymorphisms in the *VDR *pathway and factors such as vitamin D and calcium intake, blood levels of 25(OH)D, and sunlight exposure. Associations between *VDR *polymorphisms and risk of breast cancer are complex and warrant further research.

## 7. Vitamin D and Breast Cancer Survival

In terms of cancer recurrence and mortality, some studies have demonstrated an inverse relationship between vitamin D status and cancer prognosis. One large study conducted in Norway using tumor registry data from 1964 to 1992 determined that the lowest risk of cancer death occurred in those diagnosed during the seasons associated with the highest levels of vitamin D, summer or fall [169]. The authors postulated that high levels of vitamin D at the time of diagnosis and during treatment resulted in improved survival for breast, colon, and prostate cancer. 

There is a high prevalence of vitamin D deficiency among breast cancer survivors. In a multiethnic cohort of premenopausal breast cancer patients, the prevalence of vitamin D deficiency (defined as <20 ng/mL or 50 nmol/L) was 74% [84]. With supplementation of 400 IU of vitamin D_3_ daily, <15% of patients achieved sufficient blood levels of 25-hydroxyvitamin D (25(OH)D) (defined as >30 ng/mL or >75 nmol/L). Among 500 newly diagnosed breast cancer patients, 69% were found to have insufficient serum 25(OH)D concentration (defined as <32 ng/mL or <80 nmol/L) [170]. Furthermore, they found that circulating 25(OH)D levels varied by stage of disease with regional invasive disease having significantly lower vitamin D than *in situ *disease. After cancer patients were supplemented with vitamin D_3_ 8000 IU daily, mean serum 25(OH)D increased from 19.7 ng/mL (49.3 nmol/L) to 37.6 ng/mL (94 nmol/L); however, many remained in the insufficient range [171]. Another study found a 66.5% prevalence of vitamin D deficiency/insufficiency (defined as <32 ng/mL or <80 nmol/L) among 224 women with early-stage breast cancer, which was more common among non-Whites and women with later stage disease [172]. These breast cancer patients received either no vitamin D supplementation, low-dose vitamin D (1000 IU daily), or high-dose vitamin D (50,000 IU weekly) based upon their baseline serum 25(OH)D. Of note, only high-dose supplementation significantly increased 25(OH)D blood levels [172].

More recent studies have examined the association between circulating 25(OH)D levels with breast cancer recurrence and survival [82, 173–175]. Goodwin et al. used a cohort of incident breast cancer cases from Canada, 75 years of age or younger [173]. Vitamin D levels were mostly insufficient or deficient, defined as 20–29 ng/mL (50–72 nmol/L) and <20 ng/mL (<50 nmol/L), respectively. Only 24% of women in this cohort were categorized as sufficient (≥30 ng/mL or ≥75 nmol/L) [173]. In univariate analysis, both distant disease-free survival (DDFS) and overall survival (OS) were significantly worse in women with vitamin D deficiency compared to a reference group with sufficient levels (HR = 1.94, 95% CI = 1.16–3.25 and HR = 1.73, 95% CI = 1.05–2.86, resp.). However, in multivariate analysis the risk was attenuated; low vitamin D levels were only associated with DDFS (HR = 1.71, 95% CI = 1.02–2.86). Of note, the prognostic effects of vitamin D did not differ by tumor hormone receptor status. Another study in 607 postmenopausal women with early stage hormone receptor-positive breast cancer participating in a clinical trial of tamoxifen with or without octreotide found no association between baseline serum 25(OH)D and relapse-free survival [174]. In a nested case-control study within the Women's Healthy Eating and Living (WHEL) cohort, no association between serum 25(OH)D at diagnosis and breast cancer recurrence was observed; however, vitamin D intake among premenopausal women was inversely associated with recurrence [175].

Prospective studies evaluating the prognostic role of baseline circulating 25(OH)D levels for cancers of other disease sites have yielded mixed results. In a cohort study of patients with stage I-II non-small-cell lung cancer (NSCLC), serum 25(OH)D concentration at diagnosis did not correlate with clinical outcomes, except in a subset of patients with IB-IIB disease [95]. However, in patients with advanced NSCLC, no difference in survival by circulating 25(OH)D levels was observed [176]. In two studies of colon cancer patients, a significant inverse association was reported between serum 25(OH)D and overall mortality [177, 178]. Studies in prostate cancer and melanoma patients demonstrated that high serum 25(OH)D was significantly related to improved prognosis and a decreased risk of relapse and death [179, 180].

A limitation of all of these studies was that 25(OH)D blood levels were assessed at a single time point and no studies to date have evaluated change in serum 25(OH)D over time in relation to cancer prognosis. In addition, not all of these studies adjusted for age, race, body mass index, level of physical activity, and season, which can influence 25(OH)D blood levels as well as clinical outcomes [181]. Since association studies do not prove causality, low serum 25(OH)D may just be a marker of poor health among cancer patients.

Significant racial disparities in breast cancer clinical outcomes exist between African American and White women. Potential explanations for this health disparity include socioeconomic status, lifestyle factors, and access to health care; however, race is still an independent prognostic factor for breast cancer. Due to greater skin pigmentation, the mean serum 25(OH)D among African Americans is significantly lower compared to the white (16 ng/mL (or 40 nmol/L) versus 26 ng/mL (or 65 nmol/L), resp.) [14]. Vitamin D deficiency may account for some of the racial disparities between Blacks and Whites for breast cancer, colorectal cancer, cardiovascular disease, and all-cause mortality [182]. Given the high prevalence of vitamin D deficiency among African Americans, this may represent a potentially modifiable risk factor to target to reduce these health disparities.

 A number of studies have assessed VDR expression in tumor samples and *VDR *polymorphisms as prognostic markers. In studies involving patients with colorectal cancer, cholangiocarcinoma, and renal cancer, VDR tumor expression was associated with longer survival [183–185]. Among ten studies which examined the relationship between *VDR *polymorphisms and cancer prognosis, five found significant associations [165, 180, 186–188] and five did not [161, 189–192]. In breast cancer patients, the *TaqI* and *BsmI* polymorphisms were not significantly associated with prognosis [161, 191].

Vitamin D deficiency has also been linked to musculoskeletal disorders, including knee osteoarthritis [193]. In addition, severe vitamin D deficiency (serum 25(OH)D <12 ng/mL or <29 nmol/L) was associated with significantly more joint pain among postmenopausal women [194]. Joint symptoms are a common side effect of aromatase inhibitors (AIs) [195, 196], which have become the standard of care for postmenopausal endocrine-sensitive breast cancer. This AI-induced arthralgia syndrome has been associated with low 25(OH)D blood levels [197, 198]. Clinical trials have reported improvements in joint pain related to AIs among breast cancer patients who achieved circulating 25(OH)D levels of greater than 40 ng/mL (or 100 nmol/L) [198] and 66 ng/mL (or 218 nmol/L) [199]. However, results from these nonrandomized studies need to be confirmed in placebo-controlled trials. In addition, one should take into account the recent Institute of Medicine (IOM) report, which raised concerns about the potential negative health effects associated with serum 25(OH)D above 50 ng/mL (or 125 nmol/L) [11].

To determine the feasibility of conducting a randomized controlled trial of vitamin D supplementation in women with early stage breast cancer, Cescon et al. found that over 80% of these women were taking vitamin D supplements at a median dose of >1,200 IU daily and they had a median serum 25(OH)D of 34.2 ng/mL (or 85.5 nmol/L), which is already in the sufficient range [200]. Therefore, a phase III trial of vitamin D supplementation in this patient population may not be feasible.

## 8. Dosing and Toxicity of Vitamin D Supplements

Vitamin D deficiency is often defined as a circulating 25-hydroxyvitamin D (25(OH)D) level of less than 20 ng/mL (or 50 nmol/L) [201–203]. Sufficient blood concentrations of 25(OH)D generally focus on bone health, with a common definition of an optimal level of ≥30 or 32 ng/mL (≥75 or 80 nmol/L) which maximally suppresses serum parathyroid hormone (PTH). By these standards, the majority of the European and US populations are vitamin D insufficient or deficient.

A recent meta-analysis found that low vitamin D was associated with higher cancer mortality and that intake of standard doses of vitamin D supplements (with average daily doses ranging from 300 IU to 2,000 IU) was associated with a decrease in total mortality [204]. The current recommended dietary allowance (RDA) of vitamin D is 600 IU per day for those of 70 years or younger and 800 IU per day after the age of 70, but some argue that these doses are still too low to benefit public health. Based upon pooled analysis from two large studies conducted in the US and UK, women with serum 25(OH)D levels greater than 50 ng/mL (or 125 nmol/L) had a 50% lower risk of breast cancer compared to women with vitamin D deficiency [205]. Oral daily intake of 1,000 IU of vitamin D increases circulating 25(OH)D levels by about 10 ng/mL (or 25 nmol/L) [206]. However, not all individuals exhibit the same response to vitamin D supplementation. The greatest response to vitamin D supplements was observed among those who had the lowest baseline 25(OH)D blood levels [207]. Given the high prevalence of vitamin D deficiency in the general population, in order to raise serum 25(OH)D above 50 ng/mL (or 125 nmol/L), the putative target level for breast cancer risk reduction, individuals would have to consume about 3000–4000 IU daily, which is still below the current upper safety limit set by the Institute of Medicine (IOM).

In recent years, accumulating evidence suggests that daily doses as high as 10,000 IU are safe. For example, in healthy subjects at a northerly latitude, cholecalciferol 4000 IU daily for 5 months caused no harm and raised vitamin D levels to the desirable level in 88% of subjects [208]. In a review of clinical trial data on the safety of high-dose vitamin D_3_, the authors concluded that the absence of toxicity in trials conducted in healthy adults that used vitamin D doses ≥10,000 IU daily supports the selection of this value as the tolerable upper intake level [209].

However, some observational studies have found U-shaped relationships between cancer incidence rates and serum 25(OH)D levels [210–212]. Observational data suggests that the lowest all-cause mortality occurred at moderate serum 25(OH)D levels, with increased mortality risk at both low and high levels [213]. In the National Health and Nutrition Examination Survey (NHANES) III with a median followup of 8.7 years, serum 25(OH)D below 17.8 ng/mL (or 44.5 nmol/L) was associated with a 26% increase in all-cause mortality, but there was a possible increased risk above 32.1 ng/mL (or 80.3 nmol/L) [213]. Observations from a breast cancer cohort suggest that survival may be optimal for women with 25(OH)D blood levels in the range of 32–44 ng/mL (or 80–110 nmol/L) [173]. Serum 25(OH)D above this range was associated with a trend toward a higher risk of death among breast cancer patients. This U-shaped association was also observed in two cohort studies of 25(OH)D blood levels in relation to colorectal and prostate cancer risk [211, 214], and a similar nonlinear relationship was found between vitamin D status and risk of cardiovascular disease [215]. However, the statistical power to investigate risk at very high levels of serum 25(OH)D was very low.

Vitamin D toxicities, including hypercalcemia, hypercalciuria, bone demineralization, and nephrocalcinosis, are rare and generally only occur when serum 25(OH)D concentrations rise above 150 ng/mL (or 375 nmol/L) [216]. In addition, the Women's Health Initiative (WHI) randomized controlled trial found a 17% increase in the incidence of kidney stones with calcium and vitamin D supplementation compared to placebo (2.5% versus 2.1%) [217]. Therefore, the potential benefits of vitamin D supplementation for chronic disease prevention need to be carefully weighed against possible harms.

## 9. Clinical Trials of Vitamin D

Clinical trials have been conducted using the dietary supplement, vitamin D_3_ (cholecalciferol), which is a precursor to calcitriol, the active metabolite. Administering vitamin D_3_ to raise serum 25-hydroxyvitamin D (25(OH)D) levels may allow for local production of calcitriol in target tissues, with a lower risk of hypercalcemia, hypercalciuria, and kidney stones. A meta-analysis was conducted of eighteen randomized controlled trials of vitamin D supplementation (doses ranging from 300 to 2000 IU daily), which included over 57,000 participants and had fracture incidence as the main primary outcome [204]. The study demonstrated that all-cause mortality was reduced by 7% in the vitamin D group (HR = 0.93, 95% CI = 0.87–0.99). Another analysis of nineteen randomized controlled trials of vitamin D with or without calcium supplementation, including 16 trials with fracture outcomes and 3 with cancer as the primary endpoint, found insufficient evidence to support vitamin D supplementation for cancer prevention [218]. Of note, most trial participants were postmenopausal women over the age of 65 years. 

Two large randomized placebo-controlled trials in postmenopausal women examined the effect of combined calcium and vitamin D on cancer incidence. In the Women's Health Initiative (WHI) clinical trial, over 36,000 postmenopausal women were randomized to 1000 mg of calcium carbonate and 400 IU of vitamin D_3_ or matching placebo. After a mean followup of 7 years, breast and colorectal cancer incidence did not differ between the two groups [90, 219]. However, personal supplementation with vitamin D (up to 600 IU per day) was allowed, which may have dampened the ability to differentiate between the active and control arms. Women with the lowest baseline intake of vitamin D had a modest reduction (21%) in breast cancer risk with supplement use. In a nested case-control study, baseline serum 25(OH)D was inversely associated with breast cancer risk (*P* = 0.04); however, this association did not persist after adjustment for body mass index (BMI) and physical activity. A reanalysis of the data for colorectal cancer risk found that calcium and vitamin D supplementation was beneficial in women not on concurrent estrogen therapy [220]. Lappe et al. conducted another calcium and vitamin D intervention trial in 1,179 postmenopausal women using vitamin D 1100 IU daily for 4 years [94]. They found a 60% reduction in overall cancer incidence with calcium plus vitamin D compared to placebo; however, the number of cancer events was small. Neither this trial nor the WHI can distinguish between the effects of calcium and vitamin D. It remains uncertain whether targeting premenopausal women at high risk for breast cancer development or administering higher doses of vitamin D would have a protective effect on breast cancer risk.

Vitamin D and calcium are metabolically interrelated and highly correlated dietary factors which may influence breast cancer development through a variety of mechanisms [67, 221]. Calcium may also have antitumorigenic properties. A subsequent analysis of randomized controlled trials of vitamin D supplementation with or without calcium suggested that lower mortality was observed with the addition of calcium [222]. However, a population-based case-control study from Germany examined the independent and joint effects of dietary vitamin D and calcium on premenopausal breast cancer risk [57]. Breast cancer risk was inversely associated with vitamin D, but not calcium intake.

Two studies examined supplementation of vitamin D_3_ 400 IU per day in breast cancer patients concurrently treated with bisphosphonates [84, 223]. After one year of supplementation, an increase from a mean of 17 ng/mL (or 42.4 nmol/L) to 19 ng/mL (or 47.4 nmol/L) in serum 25(OH)D was observed [84]. In both study populations, approximately 60% of patients remained vitamin D deficient after supplementation [84, 223]. Two other single-arm studies examined supplementation with high doses of vitamin D in breast cancer patients [199, 224]. One study used a dose of 50,000 IU weekly of vitamin D_3_ among postmenopausal women starting adjuvant aromatase inhibitor therapy [199]. In a phase II trial in women with metastatic breast cancer, vitamin D_3_ 10,000 IU daily administered for 4 months did not have a clinical benefit for breast cancer progression [224]. In these studies, few patients experienced any toxicities from high doses of vitamin D, such as hypercalcemia, hypercalciuria, or nephrocalcinosis. These studies demonstrated that the high doses of vitamin D were well tolerated and led to a significant increase in 25(OH)D blood levels.

Calcitriol and its structural analogs have been evaluated as therapeutic agents in cancer patients. Most of the clinical trials were conducted in prostate cancer, with relatively few studies in other malignancies. A modest decrease in serum prostate-specific antigen (PSA) was observed in prostate cancer patients given calcitriol 2–2.5 *μ*g daily, indicating a decrease in disease progression [225, 226]. However, the clinical benefit was small and there was a significant increase in the incidence of renal stones [226]. To overcome these toxicities, intermittent dosing of high-dose calcitriol 3 times a week or once weekly was used [225, 227, 228]. This dosing schedule elicited a clinical response with only transient hypercalcemia and a lower risk of kidney stones. 

An alternative strategy to reduce the risk of hypercalcemia with calcitriol is to use noncalcemic vitamin D analogs. In phase I trials in a total of 58 patients with advanced breast cancer, colorectal cancer, and hepatocellular carcinoma, the calcitriol analog EB1089 (seocalcitol) caused stable disease in 6 patients for over 3 months and partial to complete remission in 2 patients [229, 230]. 

Calcitriol has been used in combination with other agents in the therapeutic setting. For example, in a phase II trial in patients with metastatic castration-resistant prostate cancer, calcitriol 12 *μ*g, 3 times per week combined with dexamethasone resulted in a 50% reduction in PSA in 28% of patients, with no hypercalcemic adverse events [228]. The addition of carboplatin to this regimen resulted in a PSA response in 13 out of 34 patients with metastatic prostate cancer [231]. The ASCENT (Androgen-independent prostate cancer study of Calcitriol ENhancing Taxotere) trials were among the largest clinical trials evaluating calcitriol in combination with chemotherapy. ASCENT I was a phase IIB randomized controlled trial in advanced prostate cancer of a standard docetaxel regimen with high-dose oral calcitriol 45 *μ*g weekly (DN-101, Novacea) [232]. The results of the primary endpoint of PSA response at 6 months was 58% among patients on DN-101 compared to 49% on placebo (*P* = 0.16). However, there was a significant improvement in the secondary endpoints of overall survival and time to progression. ASCENT II was a larger phase III trial in 953 men with metastatic castration-resistant prostate cancer with overall survival as the primary outcome [233]. The treatment arm of DN-101 with weekly docetaxel was compared to a new standard regimen of docetaxel given every 3 weeks. The trial was stopped prematurely due to an excess number of deaths in the DN-101 arm. Further analysis suggested that the asymmetric study design with docetaxel schedules led to improved survival in the control arm, rather than excess calcitriol toxicity in the treatment arm. Other phase II trials with various calcitriol formulations in advanced prostate cancer yielded mixed results [234–236].

The results of clinical trials of parent vitamin D_3_ (cholecalciferol), calcitriol, and vitamin D analogs in cancer patients and healthy individuals have yielded inconsistent results. Among prostate cancer patients, those with early recurrent disease with PSA relapse had a halting or slowing of PSA progression, whereas trials in patients with late-stage disease yielded disappointing results. Preclinical data suggests that calcitriol may have a more significant growth inhibitory effect during the early stages of cancer development. Therefore, vitamin D supplementation may have a more beneficial effect for chemoprevention in the primary prevention setting or to prevent cancer recurrence in the adjuvant setting [237].

## 10. Future Directions

More recent trials assessing moderate to high doses of vitamin D supplements to prevent cancer and other chronic diseases are currently ongoing with results due to be reported within the next 5 years (Table 2) [238]. VITAL (VITamin D and omegA-3 fatty acids triaL) is being conducted in the US and is randomizing 20,000 healthy men and women to either vitamin D_3_ 2000 IU daily, omega-3 fatty acids 1000 mg daily, the combination or placebo for 5 years. The main outcomes are cancer incidence, as well as cardiovascular disease, stroke, and diabetes. The trial is due to complete enrollment at the end of 2012, but results are not expected for several years. A New Zealand study (ViDA) comparing vitamin D_3_ 100,000 IU monthly (increased to 200,000 IU monthly in June) to placebo will likely report results in 2017. A Finnish study randomizing 18,000 elderly men and women to vitamin D_3_ 1600 IU daily, 3200 IU daily, or matching placebo will begin enrollment in 2013. In the UK, the VIDAL (VItamin D And Longevity) trial is examining an intermittent high-dose vitamin D regimen (60,000 IU monthly) on all-cause mortality. However, none of these trials are screening for low 25(OH)D blood levels at baseline for eligibility and perhaps the benefits may be limited to those with vitamin D deficiency. In addition, all of these ongoing trials are allowing personal supplement use with low-dose vitamin D (up to 800 IU daily), which will make it more difficult to distinguish between the active and control arms.

**Figure 2:**
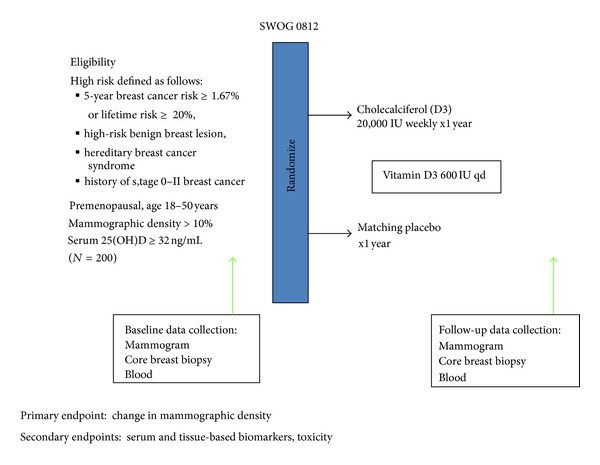
Schema for Southwest Oncology Group (SWOG) 0812 trial.

In an effort to gain a better understanding of the biologic effects of high dose vitamin D for breast cancer prevention, the Southwest Oncology Group (SWOG) is embarking on a phase IIB randomized double-blind placebo-controlled biomarker modulation study in 200 premenopausal women at high-risk for breast cancer development, based upon breast cancer risk assessment tools, presence of high-risk benign breast lesions, personal history of breast cancer, or hereditary breast cancer syndromes (Figure 2) (clinicaltrials.gov NCT01097278). Additionally, these women must have a baseline serum 25(OH)D of less than 32 ng/mL (or 80 nmol/L). They will be randomized to either vitamin D_3_ 20,000 IU weekly or matching placebo for one year. As this trial will be targeting premenopausal women with insufficient blood levels of 25(OH)D, both groups will be supplemented with a standard dose of vitamin D_3_ 600 IU daily. The primary endpoint of this trial is change in the intermediate biomarker, mammographic density. Secondarily, serial blood and benign breast tissue will be collected to assess serum and tissue-based biomarkers, respectively. Using a similar study design, the Cancer and Leukemia Group B (CALGB) is testing the effects of vitamin D 2000 IU daily for 12 months on mammographic density in 250 premenopausal women with increased mammographic density (clinicaltrials.gov NCT01224678). Both of these trials are conducting serial breast tissue sampling before and after the 1-year intervention to evaluate target tissue effects of vitamin D. For example, differential mRNA and protein expression of 1*α*-hydroxylase (*CYP27B1,* the activating enzyme) and 24-hydroxylase (*CYP24A1,* the deactivating enzyme) were demonstrated between breast cancer and benign breast tissue [28]. Differential protein expression by immunohistochemistry was noted comparing benign breast lesions to invasive breast tumors for VDR (93.5% versus 56.2%), 1*α*-hydroxylase (55.8% versus 44.6%), and 24-hydroxylase (19.0% versus 53.7%) [29]. Therefore, these trials may elucidate potential target tissue effects and mechanisms of action of vitamin D for breast cancer prevention.

## 11. Conclusions

 While a considerable amount has been learned in the past few years, much remains unknown about the use of vitamin D for the prevention or treatment of breast cancer. An inverse association between vitamin D status and breast cancer risk was apparent in the majority of studies that included serum 25(OH)D measurements. Almost all of these studies looked at a single assessment of 25(OH)D blood levels. When serum was taken prediagnostically, typically no association was found, whereas an inverse association was noted in postdiagnostic samples. 

Given the limited dietary sources of vitamin D and the increased risk of skin cancer with solar exposure, vitamin D supplementation may be the safest method to improve vitamin D status. In recent clinical trials, investigators noted an increase in levels of vitamin D with supplementation, but only in those women who were given high dose supplements. In women who were given standard doses of 400–600 IU per day, no substantial change in serum 25(OH)D was observed and the majority remained in the insufficient range [84, 90, 223]. However, if effective for breast cancer prevention, the optimal dosage of vitamin D supplementation has yet to be determined. In addition, the target population for supplementation based upon the degree of breast cancer risk, menopausal status, and baseline 25(OH)D blood levels remains unclear.

In spite of the substantial literature on the topic of vitamin D and breast cancer risk and survival, future studies need to focus on gaining a better understanding of the biologic effects of vitamin D in breast tissue. If the antitumor effects of vitamin D are confirmed in human studies, then a more accurate dosage of vitamin D for both prophylactic and therapeutic purposes needs to be established. Based upon the current literature, the Institute of Medicine (IOM) concluded that for cancer and vitamin D, the evidence was “inconsistent and insufficient to inform nutritional requirements” [11]. Therefore, the benefits of routine monitoring of serum 25(OH)D and vitamin D supplementation for breast cancer prevention or to reduce recurrence among breast cancer survivors are uncertain. Given the high prevalence of vitamin D deficiency among high-risk women and breast cancer survivors [84, 170, 239] and the relatively low toxicity and low cost of supplementation, vitamin D is a potentially modifiable risk factor to target as a strategy for breast cancer prevention and treatment.

## Figures and Tables

**Table 1:** Serum 25-hydroxyvitamin D (25(OH)D) and breast cancer risk.

Author	Year	Location	Study design	Number of cases/controls	Type of controls	Age	Comparison	OR/RR/HR (95% CI)
Abbas et al. [79–81]	2008	Germany	Case control	1394/1365	Population based	Postmenopausal	Serum 25(OH)D: <30 nM versus 30–45	OR 0.57 (0.45–0.73)
							<30 versus 45–60	OR 0.49 (0.38–0.64)
							<30 versus 60–75	OR 0.43 (0.32–0.57)
							<30 versus ≥75	OR 0.31 (0.24–0.42)

Freedman et al. [82]	2008	USA	Nested case control	1,005/1,005	PLCO cohort	Postmenopausal	Serum 25(OH)D Quintiles: <18.3, 18.3–23.5, 23.5–28.3, 28.3–33.7, ≥33.7 ng/mL	No association

Abbas et al. [83]	2009	Germany	Case control	289/595	Population based	Premenopausal	Plasma 25(OH)D: <30 nmol/L versus 30–45	OR 0.68 (0.43–1.07)
							<30 versus 45–60	OR 0.59 (0.37–0.94)
							<30 versus ≥60	OR 0.45 (0.29–0.70)

Crew et al. [89]	2009	USA	Case control	1026/1075	Population based	All ages	Plasma 25(OH)D: <20 ng/mL versus 40 ng/mL	OR 0.56 (0.41–0.78)

McCullough et al. [85]	2009	USA	Nested case control	516/516	CPS-II Nutrition cohort	All ages	Serum 25(OH)D: <50, 50–75, >75 nmol/L	No association

Rejnmark et al. [86]	2009	Denmark	Nested case control	142/420	Danish Nat'l Hospital Discharge and Danish Cancer Register	All ages	Plasma 25(OH)D: T3 (>84) versus T1 (<60 nmol/L)	RR 0.52 (0.32–0.85)
							Premenopausal, T3 versus T1	RR 0.38 (0.15–0.97)

Almquist et al. [87]	2010	Sweden	Nested case control	764/764	Malmo Diet and Cancer study (MDCS)	All ages	Serum 25(OH)D: Q2 (71–86) versus Q1 (≤70 nmol/L)	OR 0.84 (0.60–1.15)
							Q3 (87–105) versus Q1	OR 0.84 (0.60–1.17)
							Q4 (≥106) versus Q1	OR 0.93 (0.66–1.33)

Engel et al. [88]	2010	France	Nested case control	636/1272	E3N French cohort	All ages	Serum 25(OH)D: T3 (>27 ng/mL) versus T1 (<19.8)	OR 0.73 (0.55–0.96)

**Table 2:** Ongoing trials of vitamin D supplementation.

Name	Location	Study population, *N*	Dose	Outcomes	Current status	Year results expected
VITAL	US	20,000 Men, age 50+ Women, age 55+	2000 IU/d	Cancer, cardiovascular disease	Recruiting until 2012	2017
ViDA	New Zealand	5100 Age 50+	100,000 IU/mo (3300 IU/d)	Cardiovascular disease, respiratory disease, fracture	Recruiting until 2012	2017
DOHealth	Europe	2150 Age 70+	2000 IU/d	Blood pressure, fracture, infectious disease, cognition, physical function	Recruitment ongoing	2017
FIND	Finland	18,000 Men, age 60+ Women, age 65+	1600 IU/d 3200 IU/d	Cancer, cardiovascular disease, diabetes	Starting recruitment in 2013	2020
VIDAL	UK	20,000 Age 65–84	60,000 IU/mo (2000 IU/d)	Longevity and other outcomes	Ongoing recruitment of 1600 for feasibility	2020
